# Supplementary material for: GelMA Hydrogel Reinforced with 3D Printed PEGT/PBT Scaffolds for Supporting Epigenetically-Activated Human Bone Marrow Stromal Cells for Bone Repair
Source: J Funct Biomater. 2022 Apr 10;13(2):41. doi: 10.3390/jfb13020041 (PMC9036254; doi:10.3390/jfb13020041)
Supplement: Supplementary file 1 [file jfb-13-00041-s001.zip › jfb-1648445-supplementary.pdf]

Supplementary Materials

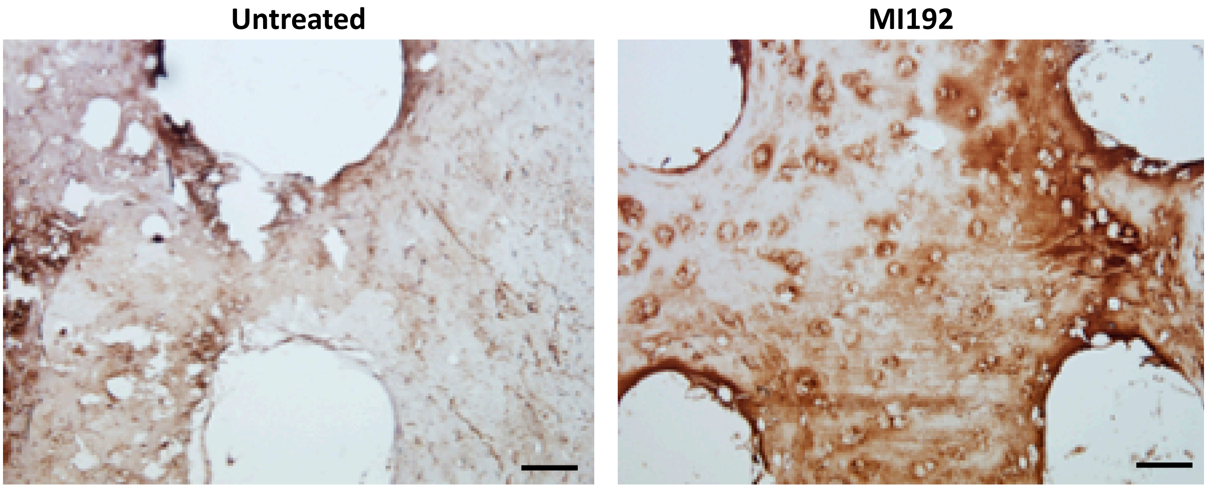

Figure S1. Immunohistochemical staining for Col1a deposition in untreated/MI192-pre-treated hBMSCs within GelMA–PEGT/PBT constructs after 6 weeks of osteogenic culture. Scale bars = 100 μm.
